# Consolidation chemotherapy after definitive concurrent chemoradiotherapy in patients with inoperable esophageal squamous cell carcinoma: a multicenter non-inferiority phase III randomized clinical trial

**DOI:** 10.1186/s12885-024-12002-5

**Published:** 2024-03-07

**Authors:** Chengcheng Fan, Xu Wang, Xiaoli Zheng, Yanan Sun, Ke Ye, Yue Jiang, Xiao Liu, Wencai Xu, Yang Liu, Yuanyuan Yang, Jinsong Liu, Qiong Jiang, Chunyu He, Xiaoyuan Wu, Xin Nie, Jingwei Zhang, Bo Tan, Wen Wang, Yougai Zhang, Zhuo Feng, Chengliang Yang, Yufei Lu, Hailong Liu, Xijuan Chen, Jing Xu, Fang Liu, Xuefeng Zheng, Jianhua Wang, Shang Wu, Guofu Chen, Yaowen Zhang, Linzhi Jin, Hong Ge

**Affiliations:** 1 The Affiliated Cancer Hospital of Zhengzhou University & Henan Cancer Hospital, Zhengzhou, 450008 China; 2 Xinyang Hospital Affiliated to Zhengzhou University & Xinyang Central Hospital, Xinyang, 464000 China; 3 Anyang Cancer Hospital, Anyang, 455000 China

**Keywords:** Esophageal squamous cell carcinoma, Consolidation chemotherapy, Definitive concurrent chemoradiotherapy

## Abstract

**Background:**

Definitive concurrent chemoradiotherapy (dCCRT) is the gold standard for the treatment of locally advanced esophageal squamous cell carcinoma (ESCC). However, the potential benefits of consolidation chemotherapy after dCCRT in patients with esophageal cancer remain debatable. Prospective randomized controlled trials comparing the outcomes of dCCRT with or without consolidation chemotherapy in patients with ESCC are lacking. In this study, we aim to generate evidence regarding consolidation chemotherapy efficacy in patients with locally advanced, inoperable ESCC.

**Methods:**

This is a multicenter, prospective, open-label, phase-III randomized controlled trial comparing non-inferiority of dCCRT alone to consolidation chemotherapy following dCCRT. In total, 600 patients will be enrolled and randomly assigned in a 1:1 ratio to receive either consolidation chemotherapy after dCCRT (Arm A) or dCCRT alone (Arm B). Overall survival will be the primary endpoint, whereas progression-free survival, locoregional progression-free survival, distant metastasis-free survival, and treatment-related toxicity will be the secondary endpoints.

**Discussion:**

This study aid in further understanding the effects of consolidation chemotherapy after dCCRT in patients with locally advanced, inoperable ESCC.

**Trial registration:**

ChiCTR1800017646.

**Supplementary Information:**

The online version contains supplementary material available at 10.1186/s12885-024-12002-5.

## Background

Esophageal cancer is the eighth most prevalent cancer and the sixth leading cause of cancer-related deaths globally [1]. In China, among all cancers, esophageal cancer has the highest morbidity and mortality rates, respectively [2]. More than 90% of patients with esophageal cancer in China demonstrate squamous cell carcinoma accounts; in contrast, esophageal adenocarcinoma is frequently observed in Western countries [3]. For nearly two decades, definitive concurrent chemoradiotherapy (dCCRT) has been the gold-standard treatment for locally advanced, inoperable ESCC [4].

The RTOG 85-01 trial established the standard pattern of dCCRT for esophageal cancer [5]. In this trial, dCCRT group patients received radiotherapy in combination with two cycles of concurrent chemoradiotherapy (CCRT; cisplatin and 5-fluorouracil), followed by two cycles of consolidation chemotherapy (cisplatin and 5-fluorouracil). These results confirmed the efficacy of dCCRT in treating locally advanced esophageal carcinoma [5]. When using combined chemoradiotherapy, subsequent studies have widely adopted the treatment regimen of two cycles of CCRT followed by another two cycles of consolidation chemotherapy. Despite significant advancements in combined treatment modalities, locoregional recurrence remains the prevailing pattern of post-dCCRT recurrence in patients with esophageal cancer, with 2-year local recurrence rates ranging from 43% to 58% [4, 6–9]. The rationale for consolidation chemotherapy in dCCRT is also based on the hypothesis that the earlier the non–cross-resistant agents are used, the higher is the likelihood of increased cancer cell death [10]. Nevertheless, the efficacy of consolidation chemotherapy after dCCRT in ESCC management has not been assessed through randomized trials. Only a few retrospective studies have compared the effectiveness of dCCRT with or without subsequent consolidation chemotherapy [11–13]. Chen et al. reported that the consolidation chemotherapy following dCCRT did not yield significant progression-free survival (PFS; 25.4 vs. 23.0 months, *P* = 0.49) and OS (35 vs. 34.6 months, *P* = 0.9) improvements in patients with ESCC [11]. However, Adenis et al. asserted that patients who receive consolidation chemotherapy after dCCRT tend to have improved median overall survival (OS; 20.1 vs. 9.9 months) and 3-year OS rate (26.4% vs. 15.5%) [13]. Nonetheless, the bias and the sample size variance in these retrospective studies have led to this issue remaining debatable. Furthermore, Wu et al. also showed that patients with ESCC did not benefit from the consolidation chemotherapy after dCCRT on both PFS and OS [12]. In the context of relevant data scarcity, a prospective non-inferiority randomized trial investigating the impact of consolidation chemotherapy after dCCRT in patients with ESCC is urgently needed.

## Rationale

To the best of our knowledge, the use of consolidation chemotherapy following dCCRT for locally advanced, inoperable ESCC was first reported over two decades previously. Furthermore, despite advancements in intensity-modulated radiotherapy technology (IMRT), dependable evidence substantiating the efficacy of consolidation chemotherapy is lacking. According to the limited evidence reported thus far, compared with dCCRT alone, consolidation chemotherapy after dCCRT does not provide a significant survival benefit in patients with locally advanced, inoperable ESCC. As such, this will be the first multicenter, prospective, phase III randomized controlled trial to provide strong evidence regarding the efficacy of consolidation chemotherapy following dCCRT in patients with inoperable ESCC.

## Methods

### Study design

**Fig. 1:**
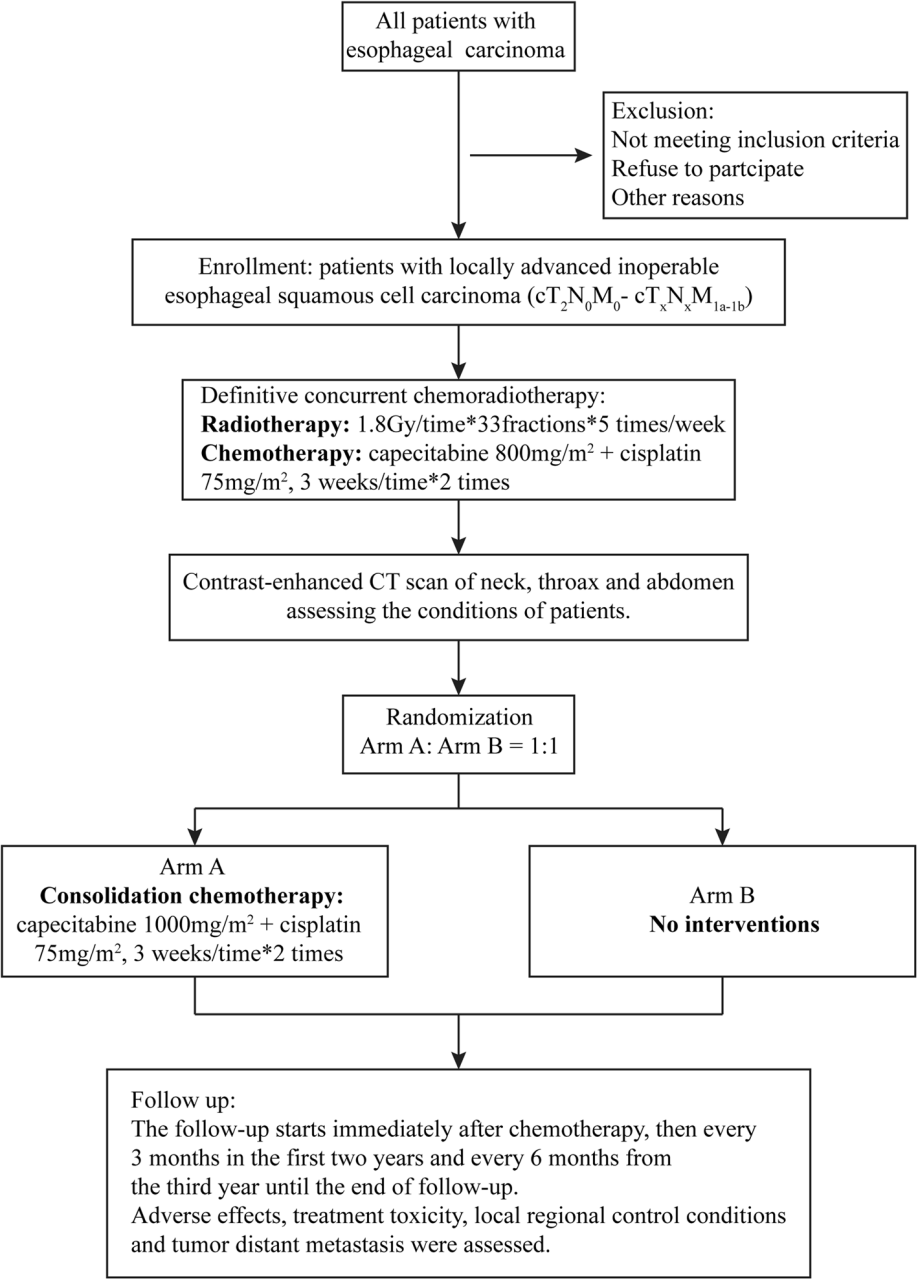
Trial diagram

This will be a prospective, two-arm, phase III randomized controlled trial aimed at assessing non-inferiority of dCCRT alone to consolidation chemotherapy following dCCRT in patients with locally advanced, inoperable ESCC. Each patient will be randomly assigned at a 1:1 ratio to receive either consolidation chemotherapy after dCCRT (Arm A) or dCCRT alone (Arm B; Fig. 1).

Randomization was stratified by tumor location (cervical *V* thoracic) and tumor stage (II *V* III *V* IV). Written informed consent will be obtained from all patients before participation in this trial. All participating medical centers are well-experienced in radiotherapy and chemotherapy delivery.

### Target population

Male and female patients with histologically proven ESCC who provide their written informed consent are eligible for this trial. The principal investigator will thoroughly evaluate and confirm that patients fulfill all the inclusion criteria.

### Sample size considerations

The sample size is based on data from a retrospective study [11] and a phase III randomized controlled trial [14]—which suggested that dCCRT yields 2-year OS rates of ≥60% and 56%, respectively. Therefore, we hypothesized that consolidation chemotherapy after dCCRT (Arm A) and dCCRT alone (Arm B) will yield 2-year OS rates of approximately 60% and 48%–65%, respectively. According to Power Analysis and Sample Size (version 15.0), these rates will be obtained with a two-sided type I error of 5% with 90% power, a type II error (β) of 20% with 15% noninferiority margin, and a dropout rate related to dCCRT or loss to follow-up of 5%. Therefore, the final sample size will be 600, with a 1:1 allocation in each arm.

### Inclusion criteria

1. Being aged 18–75 years
2. Having an Eastern Cooperative Oncology Group performance status of 0-2
3. Having histologically proven ESCC
4. Having a pretreatment stage of cT2N0M0-cTxNxM1a-1b (according to the 6th edition of AJCC/UICC TNM staging system, excluding metastasis beyond the supraclavicular or celiac lymph node and distant organ metastasis)
5. Surgically unresectable.
6. Not having prior chemotherapy or radiotherapy history and not being enrolled in other clinical trials
7. Having adequate organ function (white blood cell count ≥ 3 × 10^9^/L; neutrophil count ≥ 1.5 × 10^9^/L; hemoglobin level ≥ 90g/L; platelet count ≥ 100 × 10^9^/L; total bilirubin level ≤ 1.5 × upper limit of normal (ULN); aspartate transaminase level ≤ 2.5 × ULN; alanine transaminase level ≤ 2.5 × ULN; serum creatinine level ≤ 1.5 × ULN)
8. Having a life expectancy of ≥3 months
9. Providing written informed consent.

### Exclusion criteria

1. Having a past or current history of another malignant disease, except completely treatable nonmelanoma skin cancer and cervical carcinoma in situ (with PFS ≥ 3 years).
2. Having an uncontrolled cardiac disease (e.g., myocardial infarction within the previous 6 months and coronary artery disease), diabetes, or hypertension.
3. Having a severe mental disorder
4. Being pregnant or lactating
5. Having a tumor invading the airway or aorta
6. Having complete obstruction due to esophageal cancer, signs of esophageal perforation or bleeding, or already developing perforation and bleeding
7. Receiving oral or intravenous corticosteroids
8. Having dysfunction in major organs, such as the liver, kidneys, heart, and lungs, immune deficiency, or active infection
9. Having other possibly ineligible conditions based on the researchers’ judgment

### Exit criteria

1. Cancer progression
2. Request to withdraw from the trial
3. Development of grade 4 or higher nonhematologic toxicity (according to Common Terminology Criteria for Adverse Events (CTCAE; version 4.0), which could not reduce grade 2 or after treatment) or development of serious adverse events.
4. Interruption in radiotherapy for >2 weeks for any reasons
5. No treatment initiation for 4 weeks after enrollment
6. Development of possibly ineligible conditions based on the researchers’ judgment

### Study interventions

#### Chemotherapy

CCRT (Both arms)

All patients will receive two cycles of chemotherapy (cisplatin: 75 mg/m^2^/day, IVGTT, day 1; capecitabine: 800 mg/m^2^/day, bid, PO, d1–14) every 3 weeks during radiotherapy.

Consolidation chemotherapy (Arm A)

After CCRT, patients in arm A will receive two cycles of consolidation chemotherapy (cisplatin: 75 mg/m^2^/day, IVGTT, day 1; capecitabine: 1000 mg/m^2^/day, bid, PO, d1–14) every 3 weeks.

Observation group (Arm B)

Patients in arm B will receive no interventions.

#### Radiotherapy

The treatment plan is shown in Fig. 1. Radiation will be delivered with ≥6-MV photons 5 days per week to administer a total dose of 59.4 Gy in 33 fractions. All patients will be placed in the treatment position in an individualized immobilization device, and IMRT will be used.

The definition of volumes will be in accordance with the Report 50 (1993) and Report 62 (1999) of the International Commission on Radiation Units & Measurements (ICRU). The gross target volume (GTV) is defined as the gross demonstrable location and extent of the tumor, detected through endoscopic ultrasound, barium swallow x-ray, or computed tomography (CT) (whichever is larger). A regional lymph node with a diameter of ≥1 cm (for cervical, mediastinal, or celiac lymph nodes) or 0.5 cm (for tracheoesophageal groove lymph nodes) or that proven to be metastatic through positron emission tomography (PET)–CT will be defined as a GTVnd. Involved-field irradiation will be adopted when defining the clinical target volume (CTV). The CTV encompasses the GTV and the region draining the lymphatics, which will be defined as follows: a 3-cm craniocaudal margin, a 0.5–0.8-cm lateral margin beyond the GTV, and a GTVnd with a 1–1.5cm margin, including the metastatic lymph nodes. The PGTV is at 1 cm craniocaudally beyond the GTV and 0.5cm radially and the GTVnd. The planning target volume (PTV) will be defined as the CTV plus a 0.5–0.7cm expansion. Patients will be treated typically with daily fractions of 1.8 Gy to a total initial dose of 50.4 Gy to the PTV, followed by a cone down of 9 Gy to a total dose of 59.4 Gy to the PGTV.

The treatment plan will consider normal organ dose constraints as follows: The volume of lung tissue receiving ≥20 Gy should be <28% of the total lung volume, and the mean dose has to be <18 Gy. For the spinal cord, the maximal point dose should be <45 Gy. The volume of the heart receiving ≥30 Gy radiation must be <40% of the total heart volume, and the mean dose should be <26 Gy. The PTV and the organ at risk will be assessed using a dose–volume histogram.

### Dose modifications

#### Radiotherapy interruption

In patients who develop grade 3 or higher hematological or nonhematological toxicity, radiotherapy will be delayed until the toxicity grade decreases to ≤2. At most, a 2-week delay will be permitted. Patients with a longer delay will be excluded from the trial.

#### Chemotherapy interruption

Chemotherapy dose modifications will be based on the most severe toxicity in the patients' last cycle. In patients who develop grade 2 or higher hematological toxicity, chemotherapy will be delayed until the toxicity grade decreases to ≤1. At most, a 2-week delay will be permitted. Patients with a longer delay will be excluded from the trial and asked to discontinue chemotherapy.

Chemotherapy dose modifications may be required in the following situation: a patient develops grade 4 hematological toxicity or grade 3 or higher nonhematological toxicity, but the toxicity grade decreases to ≤1 within 2 weeks before the next cycle. Patients who remain ineligible after this period will be asked to discontinue chemotherapy.

If modification is necessary, cisplatin and capecitabine doses will be reduced to 75% of the planned dose. Dose modifications may be made at most twice; if more than two modifications are required, chemotherapy will be terminated.

### Study objectives

The main objective of this trial is to compare OS in ESCC patients treated with or without consolidation chemotherapy after dCCRT. Our secondary objectives are to compare PFS, locoregional PFS (LPFS), distant metastasis-free survival (DMFS), and treatment-related toxicity.

OS is defined as the time from the date of dCCRT initiation until the date of death. PFS is defined as the time from the date of dCCRT until the date of first progression or death. LPFS is defined as the time from the date of dCCRT until the date of first locoregional progression (a primary tumor or locoregional lymph nodes) or death. DMFS is defined as the time from the date of dCCRT until the date of first distant metastasis (nonregional lymph nodes or a distant organ) or death.

### Pretherapeutic assessments

Before administering any treatment, we will perform routine examination and record its results for every potential patient. The routine examination will include the following:

1. A physical examination, as well as vital sign and medical history taking
2. Karnofsky performance status and Eastern Cooperative Oncology Group performance status
3. Standard laboratory tests (routine complete blood count and blood biochemistry), pulmonary function test, and electrocardiography
4. Upper digestive tract endoscopy with biopsy
5. CT of the thorax or PET–CT; magnetic resonance imaging (MRI) of the esophagus, endoscopic ultrasound of the esophagus, or both; brain MRI (if needed); whole-body bone emission CT (if required); or CT or ultrasound of the neck and abdomen
6. European Organisation for Research and Treatment of Cancer quality of life

### Assessments during the treatment phase

The outcomes of physical examinations, vital signs, routine complete blood count, blood biochemistry, and radiotherapy-related pulmonary and esophageal toxicity will be recorded weekly during the dCCRT period, according to CTCAE (version 4.0). Changes in esophageal wall thickness and lymph node size (if needed) will be evaluated through contrast-enhanced CT of the neck, thorax, and abdomen. Changes in the esophageal tract will be assessed through contrast-enhanced CT of the upper gastrointestinal tract. The residual primary tumor will be evaluated through upper endoscopy followed by biopsy. Local tumors, locoregional lymph nodes, and distant metastases will be assessed through PET–CT. After 2–6 weeks of dCCRT, patients without disease progression will be randomized at a 1:1 ratio to arm A or B by a central randomization center; moreover, patients demonstrating disease progression will be excluded from the study.

### Assessments during the follow-up phase

After completion of the study treatment, patients will be followed up every 3 months for the first 2 years and then every 6 months from the third year onward. All patients will be followed up until death or at least 3 years after treatment.

### Statistical analysis

Data analyses will be performed according to the intention-to-treat principle in all randomized patients. Between-group comparisons will be performed using chi-squared and Fisher’s exact test for categorical parameters and Student’s *t* test for normally distributed continuous variables or the Mann–Whitney *U* test or analysis of variance for nonnormally distributed continuous variables. Survival rates will be calculated using the Kaplan-Meier method, and comparisons between the groups will be performed using the log-rank test. The difference will be considered statistically significant at a *P* value of <0.05.

### Funding and current status

Our study has been approved by the Medical Ethics Committee of Henan Cancer Hospital (2018087) and supported by the Health Commission of Henan Province (182106000062). Moreover, the funding body has peer-reviewed the study protocol.

Our study commenced on July 27, 2018. Currently, it remains in the patient recruitment stage. Since August 1, 2018. 200 patients have been recruited.

## Discussion

Numerous trials have reported that compared with radiotherapy alone, dCCRT can significantly reduce treatment failure and prolong survival in patients with esophageal cancer [4–6, 14–16]. However, a majority of these trials have primarily included populations from Western countries, where esophageal adenocarcinoma is more prevalent than ESCC. In contrast, ESCC is the predominant histological type of esophageal cancer in China [2]. Therefore, exploring alternative treatment approaches specifically targeting ESCC is warranted.

Consolidation chemotherapy is defined as the prolongation of chemotherapy duration by the administration of additional drugs at the end of a defined number of initial chemotherapy cycles, after the achievement of maximum tumor response, in individual patients [17]. According to the RTOG 85-01 trial, the most adopted dCCRT scheme includes two cycles of consolidation chemotherapy after dCCRT [5]. However, the specific role and rationale for incorporating consolidation chemotherapy remain undefined; nevertheless, incorporating consolidation chemotherapy may reduce the likelihood of distant metastases and ultimately improve OS. Thus far, only a few retrospective studies have focused on consolidation chemotherapy. For instance, Chen et al. and Wu et al. have demonstrated that consolidation chemotherapy does not yield significant improvements in disease control or PFS in patients with ESCC. In contrast, Adenis et al. suggested that patients who undergo consolidation chemotherapy tend to have prolonged OS [11–13]. Furthermore, Wang et al. compared the potential survival advantages of consolidation chemotherapy and dCCRT alone in patients diagnosed as having unresectable esophageal cancer; the authors revealed a short-term survival benefit and a reduced likelihood of distant metastasis [18]. Similarly, Lin et al. and Xia et al. observed that consolidation chemotherapy is strongly associated with prolonged OS in ESCC patients undergoing dCCRT [19, 20]. However, the results observed across the aforementioned studies have been heterogeneous; therefore, additional prospective randomized controlled trials validating the benefits of consolidation chemotherapy in patients with ESCC are needed urgently.

The incidence rate of local recurrence among esophageal cancer patients who undergo dCCRT can be 43%–58%, and this recurrence can significantly reduce the patients' long-term survival rates [7–9]. Recent studies have suggested that LPFS of ESCC patients receiving dCCRT with a higher radiation dose (≥59.4Gy) and those receiving dCCRT with a standard radiation dose (50 Gy/2 Gy or 50.4 Gy/1.8 Gy) is comparable [21, 22]. The standard radiation dose for dCCRT is 50 Gy, as indicated in the RTOG 85-01 trial [5]. However, Chang et al. noted that a higher dose of radiation (≥60 Gy) may prolong survival [23]. This finding was further supported by another study [24]—which also indicated that a higher dose may enhance local control. Nevertheless, recent randomized studies have demonstrated that in dCCRT for ESCC, the standard radiation dose (50 Gy/2 Gy or 50.4 Gy/1.8 Gy) has an effect similar to that of a higher radiation dose (≥59.4 Gy/1.8 Gy) but with relatively less treatment-related toxicity [21, 22, 25, 26]. Consequently, in future clinical practice, the standard radiation dose should be recommended for dCCRT in patients with ESCC.

Although several studies have analyzed the effects of different CCRT regimens, none have demonstrated considerably improved treatment efficacy or substantially decreased toxicity [15, 16, 27–30]. 5-Fluorouracil plus cisplatin has been used as a standard regimen in many dCCRT studies after the RTOG 85-01 trial [5, 6, 31–33]. This chemotherapy regimen achieves a curative effect but with serious treatment toxicity, such as severe myelosuppression and mucositis [5]. In three trials (including the SCOPE1 trial), capecitabine, an oral prodrug of 5-fluorouracil, demonstrated a high complete remission rate with acceptable toxicity [28, 29]. Therefore, capecitabine plus cisplatin may be a more acceptable candidate for subsequent dCCRT trials [14, 34, 35]. Some trials have also explored the efficacy of other chemotherapy regimens in dCCRT, such as paclitaxel plus carboplatin and the FOLFOX regimen [15, 17, 36] and 5-fluorouracil plus paclitaxel [30]. However, none of the aforementioned regimens demonstrates relatively high efficacy or relatively less toxicity.

Even though dCCRT remains the predominant treatment modality for esophageal cancer patients, high-level evidence regarding the use of consolidation chemotherapy for patients with ESCC is highly insufficient. To date, no prospective study has compared the outcomes of dCCRT with or without consolidation chemotherapy in patients with ESCC. Nevertheless, the current prospective randomized controlled trial will aid in understanding whether consolidation chemotherapy after dCCRT yields relatively superior benefits in patients with locally advanced, inoperable ESCC.

In conclusion, our study will be a multicenter, prospective, open-label, phase III randomized controlled trial to investigate the efficacy and safety of consolidation chemotherapy following dCCRT in patients with inoperable ESCC. The study will make valuable contributions to the evidence regarding the effects of consolidation chemotherapy for esophageal cancer in China.

### Supplementary Information

**Supplementary Material 1.**

## Data Availability

This article has used no dataset. Therefore, no additional data files are available.

## Abbreviations

dCCRT: Definitive concurrent chemoradiotherapy
ESCC: Esophageal squamous cell carcinoma
PFS: Progression-free survival
LPFS: Locoregional PFS
DMFS: Distant metastasis-free survival
OS: Overall survival
IMRT: Intensity-modulated radiotherapy technology
AJCC: American joint committee on cancer
UICC: International union against cancer
ULN: Upper limit of normal
CTCAE: Common Terminology Criteria for Adverse Events
PO: Peros
IVGGT: Intravenously guttae
ICRU: International Commission on Radiation Units & Measurements
GTV: Gross target volume
CTV: Clinical target volume
PTV: Planning target volume
CT: Computed tomography
MRI: Magnetic resonance imaging
