# Supplementary material for: Consolidation chemotherapy after definitive concurrent chemoradiotherapy in patients with inoperable esophageal squamous cell carcinoma: a multicenter non-inferiority phase III randomized clinical trial
Source: BMC Cancer. 2024 Mar 7;24:321. doi: 10.1186/s12885-024-12002-5 (PMC10921589; doi:10.1186/s12885-024-12002-5)
Supplement: Supplementary file 1 — Supplementary Material 1. [file 12885_2024_12002_MOESM1_ESM.docx]

**Treatment Schedule**

| **Treatment Phase** | **Screening** | **dCCRT treatment (1^st^ week)** | **dCCRT treatment (2^nd^ week)** | **dCCRT treatment (3^rd^ week)** | **dCCRT treatment (4^th^ week)** | **dCCRT treatment (5^th^ week)** | **dCCRT treatment (6^th^ week)** | **Consolidation chemotherapy** | **Follow up** |
| --- | --- | --- | --- | --- | --- | --- | --- | --- | --- |
| Time point | <14 days before dCCRT | At day 1 of each week | | | | | | | Starting 1 month after consolidation therapy or dCCRT, every  3/6 months^[[1]](#endnote-1)^* |
| Informed consent | x |  |  |  |  |  |  |  |  |
| Demography^[[2]](#endnote-2)^ | x |  |  |  |  |  |  |  | x |
| Medical history | x |  |  |  |  |  |  |  | x |
| Vital sign (P, R, T, BP) | x | x | x | x | x | x | x | x | x |
| Physical examination^[[3]](#endnote-3)^ | x | x | x | x | x | x | x | x | x |
| ECOG performance score | x |  |  |  |  |  |  |  | x |
| CT thorax/abdominal | x |  |  |  |  |  |  | x | x^[[4]](#endnote-4)^ |
| Endoscopic ultrasound | x |  |  |  |  |  |  |  | x |
| Upper GI endoscopy | x |  |  |  |  |  |  |  | x |
| Histopathology report | x |  |  |  |  |  |  |  | x |
| ECG | x |  |  |  |  |  |  |  | x |
| Pulmonary function | x |  |  |  |  |  |  | x | x |
| Blood routine^[[5]](#endnote-5)^ | x | x | x | x | x | x | x | x | x |
| Biochemistry ^[[6]](#endnote-6)^ | x |  | x |  | x |  | x | x | x |
| Randomization |  |  |  |  |  |  |  | x |  |
| Adverse events and complications | x | x | x | x | x | x | x | x | x |
| Quality of life (EORTC QLQ-C30, OES18) | x | x |  |  |  |  |  | x | x |

1. * The first follow-up visit is performed 1 months after consolidation chemotherapy or dCCRT. From then on, follow-up visits are carried out every 3 months (+/- 7days) in the first two years of follow-up and every 6 months (+/- 7days) from the third year after treatment until the end of follow-up (min. 3 years). [↑](#endnote-ref-1)
2. Demography includes sex, age, height, race, ethnicity, job category, allergy and so on [↑](#endnote-ref-2)
3. Physical examination includes, but is not limited to, cardiovascular, gastrointestinal, hepatobiliary, respiratory, musculoskeletal, skin, neurological, genitourinary/renal and other organ systems. [↑](#endnote-ref-3)
4. Contrast-enhanced CT of Thorax/Abdomen is carried out regularly at follow-up visits. Further diagnostic investigations, including PET-CT, Upper endoscopy are performed only if suspected to be recurrence or metastasis at the discretion of the investigator/treating physician. [↑](#endnote-ref-4)
5. Blood routine includes hemoglobin, total red count, total white blood count, platelet count, and a differential white count including neutrophils, lymphocytes, monocytes, eosinophils and basophils. [↑](#endnote-ref-5)
6. Biochemistry includes (but is not limited to) AST, ALT, total bilirubin, blood glucose, serum creatinine, sodium, potassium. [↑](#endnote-ref-6)
